# Infective Endocarditis After Endoscopic Stricture Dilation in Crohn's Disease

**DOI:** 10.14309/crj.0000000000001377

**Published:** 2024-06-20

**Authors:** André Gonçalves, Sandra Barbeiro, Carina Leal, Antonieta Santos, Helena Vasconcelos

**Affiliations:** 1 Department of Gastroenterology, Centro Hospitalar de Leiria, Leiria, Portugal

**Keywords:** endoscopic balloon dilation, infective endocarditis, Crohn's disease, stricturing disease

## Abstract

Stricture formation is common in Crohn's disease, and endoscopic intervention plays an increasingly important role in managing these strictures. A 61-year-old man with biological aortic prosthesis and a 30-year history of ileocolonic stricturing Crohn's disease, managed with azathioprine and infliximab, presented with marked occlusive symptoms. Colonoscopy revealed a descending colon stricture, prompting endoscopic balloon dilation. At the time of the procedure, no prophylactic antibiotic was given. Subsequently, he developed Streptococcus gallolyticus endocarditis, necessitating aortic valve replacement. The authors present a case of late *Streptococcus gallolyticus* endocarditis associated with endoscopic balloon dilation of a Crohn-related colonic stricture.

## INTRODUCTION

Crohn's disease (CD) is an inflammatory bowel disease (IBD) characterized by chronic inflammation involving any part of the gastrointestinal (GI) tract. It can present as stricturing, penetrating, or nonstricturing/nonpenetrating phenotypes. Stricture formation is frequent in CD, and endoscopic treatment is becoming increasingly important. There are several complications of endoscopic procedures, infective endocarditis being one of the rarest.^1–3^ The authors present a case of infective endocarditis following a dilation of a Crohn-related colonic stricture.

## CASE REPORT

**Figure 1.**
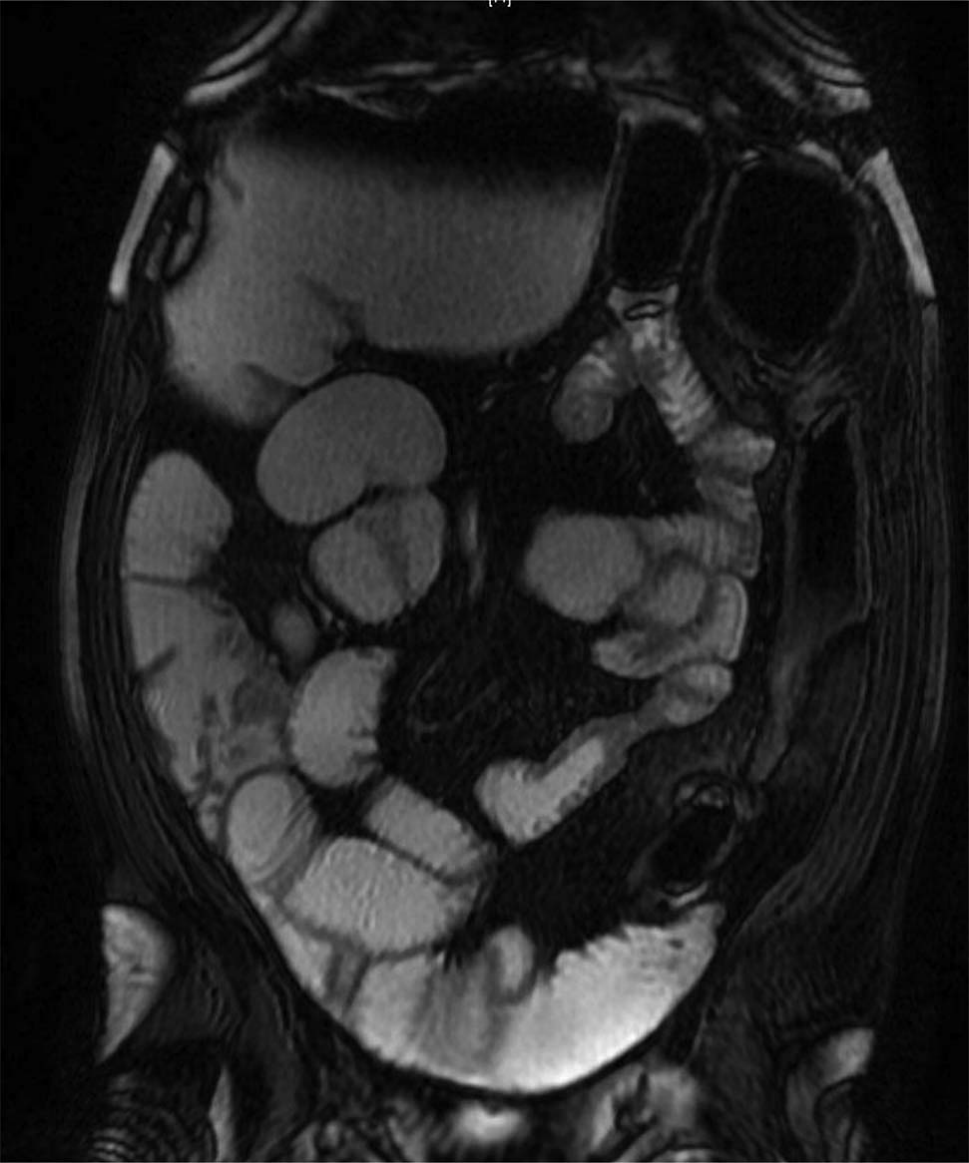
Magnetic resonance image showing a 4 cm long colon's fibrotic stenosis.

**Figure 2.**
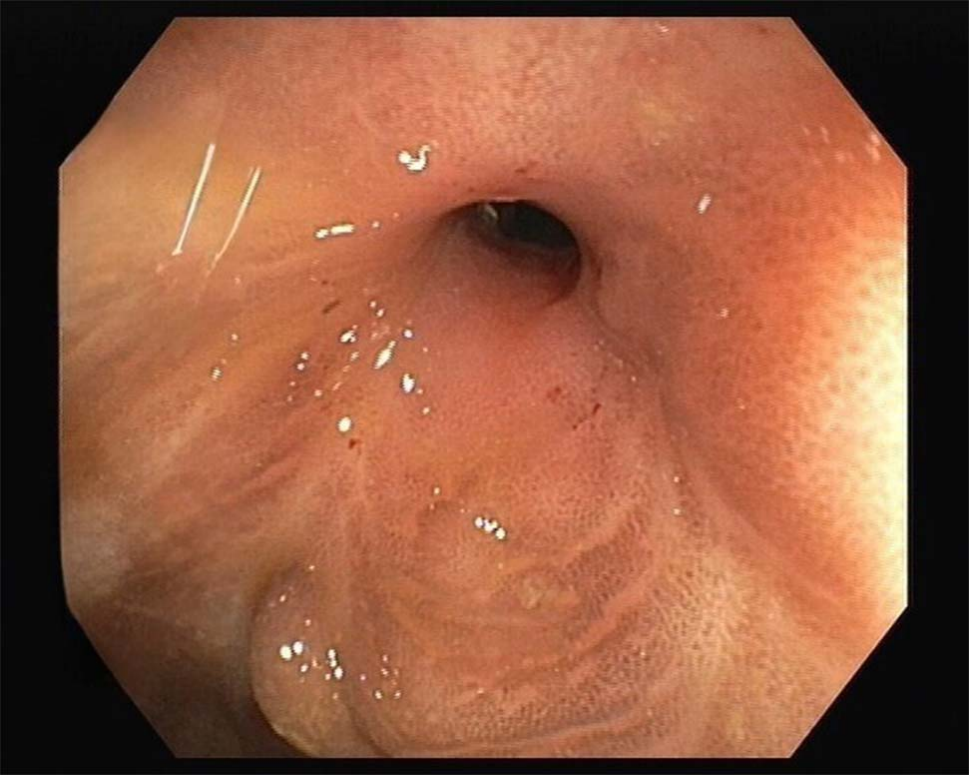
Colonoscopy (on admission) revealing a nontransversable and nonulcerated stricture in the descending colon.

A 61-year-old man was admitted to the gastroenterology ward twice in a couple of months because of CD exacerbation with symptoms of intestinal subocclusion. He had a 30-year history of ileocolonic stricturing CD (Montreal score A2L3B2), along with long-term, intermittent, mild obstructive symptoms. His medical history included aortic insufficiency with implantation of a biological aortic prosthesis in 2018. Long-term maintenance therapy included azathioprine 2 mg/kg/d and infliximab 5 mg/kg (8/8 weeks). Serum infliximab levels were at the therapeutic range, and anti-infliximab antibodies were absent. His last magnetic resonance image described a 4 cm long left colon mainly fibrotic stenosis, with marked distension of the transverse colon and no other findings (Figure 1). Colonoscopy on admission revealed a nonulcerated stricture in the descending colon. The stenosis was not transversable (Simple Endoscopic Score for CD of 3 points); biopsies ruled out malignancy. The patient improved with medical therapy, so invasive procedures were postponed, yet mild, insidious symptoms remained (Figure 2). A joint medical and surgical assessment was performed and an elective through-the-scope endoscopic ballon dilation (EBD) of the stenosis was scheduled. A first dilation up to 10 mm followed by another (after 4 weeks) up to 12 mm were performed without immediate complications. At the time of the procedure, the patient was under maintenance combination therapy, and no prophylactic antibiotic was given.

**Figure 3.**
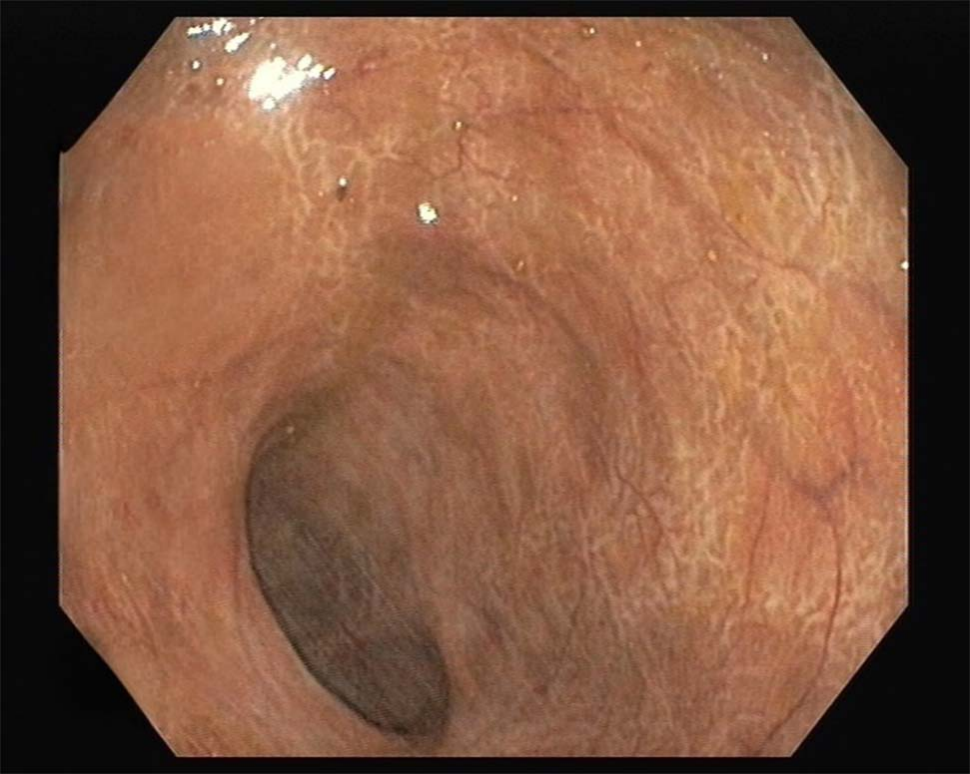
Colonoscopy (after endoscopic ballon dilation): Colon upstream of the stenosis remarkably dilated and the mucosa with several areas of scarring.

**Figure 4.**
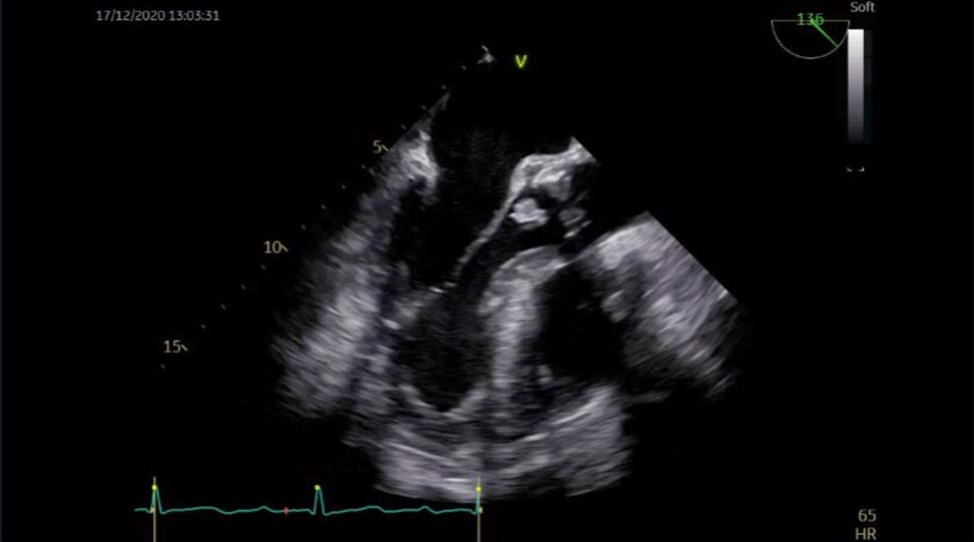
Transthoracic echocardiogram described a mobile hyperechogenic filiform image with 9.2 × 7.5 mm in diameter, in dependence of the right coronary cuspid.

After the second dilation, the stenosis was transposable with a conventional gastroscope. The colon upstream of the stenosis was remarkably dilated, and the mucosa had several areas of scarring, with no other findings (Simple Endoscopic Score for CD score of 1 points) (Figure 3). As symptoms resolved, no further endoscopic dilatation was scheduled. A couple of weeks after the last procedure, the patient was admitted to the emergency department due to persistently high fever. Admission laboratory work revealed an elevated inflammation profile with leukocytosis (14, 500 μL) and neutrophilia (88%). After a thorough evaluation, a transthoracic echocardiogram was performed, which showed a “mobile hyperechogenic filiform image in dependence of the right coronary cuspid (suggestive of vegetation)” (Figure 4). A diagnosis of endocarditis was made. Antibiotic therapy was initiated and adjusted after isolation of *Streptococcus gallollyticus* in blood cultures. As ultrasonographic findings worsened, surgical aortic valve prosthesis replacement was necessary. The surgical intervention and postoperative period were uneventful. At 16-month follow-up, the patient remains asymptomatic. Further stricture dilation was deemed unnecessary as technical and clinical success was achieved.

## DISCUSSION

The authors report a case of late endocarditis due to *S*.* gallollyticus* in an aortic bioprosthetic valve, possibly related to an endoscopic procedure in an immunocompromised patient with CD.^4^ The stricturing phenotype in CD often leads to occlusive symptoms.^1,5–7^ EBD is now considered as first-line therapy for noncomplex strictures that are less than 4–5 cm in length, without concurrent perforation, abscesses, or adjacent fistulae.^2,8–10^ EBD demonstrates a technical success rate of 89% and a clinical success rate of 81%. It is considered a safe procedure, with complications occurring in less than 3% of cases and a low risk of infection.^5,11^

Although antibiotic prophylaxis has been suggested in high-risk cardiac conditions, evidence for this recommendation in endoscopic procedures is sparse and of low quality.^3,4^ Infections following endoscopic procedures are presumably the result of bacteremia induced by the translocation of endogenous bacteria via mucosal trauma. The rates of bacteremia following GI procedures are generally low (5%–10%) and rarely result in clinically evident infection.^8–10^ Interestingly, Shah-Khan et al reported a similar proportion of infective endocarditis due to gram-negative bacteria in patients with inflammatory bowel disease and the general population, concluding that infective endocarditis is unrelated to the risks of bacteremia from endoscopy. Moreover, the potential benefits of prophylaxis need to be outweighed against the risks of resistance, anaphylaxis and *Clostridioides difficile* infection.^12^

According to European Society of Cardiology Guidelines on Prevention, Diagnosis, and Treatment of Infective Endocarditis, no routine prophylaxis for GI procedures is indicated.^4^

The incidence of infective endocarditis in patients with IBD has been rising. While many causes may concur to this, immunosuppression is likely to play a significant role. Overall, the Crohn's Therapy, Resource, Evaluation, and Assessment Tool (TREAT) registry has demonstrated that patients with CD had increasing rates of serious infection when receiving infliximab and prednisone. We report a case of combination therapy, and the risk of serious infections is also known to be higher in these patients.^13^

Based on a 2020 published consensus statement from the Global Interventional Inflammatory Bowel Disease Group, antibiotic prophylaxis in colonoscopy of strictures in Crohn's disease should be considered in patients who are at risk of perforation and peritonitis. The risk factors proposed by the authors include corticosteroid use; malnourishment; prolonged procedures; several mucosae or transmural inflammation; multiple angulated strictures and strictures in patients with ileostomies; and diverted colon, rectum, or ileal pouch.^8^

A 2015 literature review by the American Society for Gastrointestinal Endoscopy found no cases of infective endocarditis following endoscopic procedures, none following EDB of a colonic stricture. GI procedures present a low risk of bacterial endocarditis, and there is only a weak association of GI procedures with infective endocarditis, as shown by the lack of data supporting a causal link.^3^

Despite the cardiac history and long-term immunosuppressive therapy, the decision not to administer prophylactic antibiotics in this case was based on a thorough, individualized assessment of the patient. Furthermore, as mentioned above, the patient had no risk based on the consensus from the Global Interventional IBD Group.^8^ While EBD has demonstrated technical and clinical success and is considered a safe procedure, it is essential to carefully select patients. The risk of infective endocarditis following endoscopic procedures, including EBD, remains a subject of debate.

## DISCLOSURES

Author contributions: A. Gonçalves wrote the manuscript. S. Barbeiro, C. Leal revised the paper critically for important intellectual content. A. Santos and H. Vasconcelos participated in patient management during hospitalization. All authors read and approved the final version of the manuscript. A. Gonçalves is the article guarantor.

Financial disclosure: None to report.

Previous presentation: This paper was presented as oral communication at the European Society of Gastrointestinal Endoscopy Days 2023 Congress; April 22, 2023; Dublin, Republic of Ireland.

Informed consent was obtained for this case report.

The project was subjected to the standards of good clinical practice and always complied with the ethical precepts of the Helsinki Declaration. Written informed consent was obtained from the patient for publication of this case report and any accompanying images.
